# A Decentralized Signcryption Scheme Based on CFL

**DOI:** 10.3390/s25061773

**Published:** 2025-03-12

**Authors:** Leyi Shi, Mengting Liu

**Affiliations:** 1 College of Computer Science and Technology, China University of Petroleum, Qingdao 266580, China; by1907010303@s.upc.edu.cn; 2 Qingdao Guochuang Intelligent Appliance Research Institute Co., Ltd., National Innovation Institute of High-End Smart Appliances, Qingdao 266101, China

**Keywords:** signcryption, CFL, provable security, random oracle model, SM2

## Abstract

The rapid advancement of quantum computing technology poses a significant threat to conventional public key cryptographic infrastructure. The SM2 (state key cryptography algorithm no. 2) elliptic curve public key cryptographic algorithm, which adopts elliptic curve cryptography, has demonstrated strong resistance to quantum attacks. However, existing signcryption schemes remain vulnerable due to their reliance on a single certification authority (CA) managing all keys. The cryptography fundamental logics (CFL) authentication process eliminates the need for third-party involvement, achieving decentralized authentication and reducing the burden on certificate generation centers. Therefore, a decentralized signcryption scheme based on CFL was proposed using the SM2 national cryptographic algorithm. Unlike traditional signcryption schemes, this approach does not depend on the certification authority’s private key during the public–private key generation process. This innovation helps avoid risks associated with certification authority private key leakage and ensures decentralized characteristics. The proposed scheme was rigorously verified under the random oracle model (ROM) and based on the complexity assumption of the elliptic curve Diffie–Hellman (ECDH) problem. The theoretical analysis and experimental results demonstrate that compared to traditional methods, the proposed scheme exhibits higher efficiency in communication and computation. Specifically, the proposed scheme reduces computational overheads by approximately 30% and communication overheads by approximately 20% in practical working environments. These quantitative improvements highlight the scheme’s promising application prospects and practical value.

## 1. Introduction

Confidentiality and authentication are two crucial indicators in cryptography, and the newly developed signcryption technology has adhered to these principles in its evolution. Early cryptographic research explored and applied these two indicators separately, such as confidentiality in public-key cryptosystems and authentication in digital signatures. However, with societal advancement, the demands of electronic operations (such as email and electronic payments) require signcryption technology to simultaneously satisfy both key indicators while keeping pace with the times. Addressing this need, researchers initially proposed the “sign-then-encrypt” implementation path, but this approach was not widely adopted due to high computational and communication costs. Subsequently, in 1997, Zheng et al. [1] proposed integrating both functions into one unified approach, efficiently satisfying both key indicators simultaneously. This method became known as public-key signcryption.

Signcryption technology offers the advantages of cost-effectiveness, high operational efficiency, enhanced security, and simplified design implementation. In recent years, with increasing security awareness, signcryption technology has become a focal point of research among scholars and engineers due to its dual capability of ensuring both confidentiality and authentication. Based on the maturity of existing research theories and scheme designs, it has gradually achieved mass production and widespread application (such as in electronic payments, mobile agent security, and firewalls), yielding favorable practical results. Through these practical applications, signcryption technology has gradually crossed over from cryptography to network security and other fields, becoming a crucial topic in related domains.

Simultaneously, with the rapid development of quantum computing technology, traditional public-key cryptosystems face unprecedented security challenges [2]. The SM2 algorithm [3], independently developed in China, based on elliptic curve cryptography principles, demonstrates significant advantages in resisting quantum computing attacks. The SM2 algorithm utilizes the discrete logarithm problem in elliptic curve point groups and provides higher security strength than the RSA algorithm. Furthermore, the SM2 algorithm employs shorter key lengths, typically 256 bits, whereas RSA typically requires 2048 bits or more. These shorter key lengths enable reduced storage and transmission data volume while maintaining equivalent security levels, thereby improving efficiency. The SM2 algorithm’s computational complexity is lower than that of RSA, offering advantages in processing speed, particularly in scenarios requiring rapid encryption and decryption. Most importantly, due to its elliptic curve-based characteristics, the SM2 algorithm is considered to possess some degree of resistance against quantum computing attacks.

Traditional public key infrastructure (PKI) relies heavily on centralized certificate authorities (CAs) to manage and validate digital certificates, which bind public keys to identities. This centralized model, while widely adopted, introduces several inherent weaknesses. Firstly, it creates a single point of failure and a potential target for attacks. Compromise of a CA’s private key can have catastrophic consequences, potentially invalidating the entire trust framework and enabling widespread impersonation and data breaches. Secondly, the CA model can be cumbersome and inefficient, particularly in dynamic and large-scale distributed systems. Certificate generation, distribution, and revocation processes can introduce latency and complexity, hindering the agility and scalability of applications. Furthermore, the reliance on a central authority can be antithetical to the principles of decentralization and autonomy, which are increasingly valued in modern distributed systems, such as blockchain technologies, Internet-of-Things (IoT) networks, and vehicular ad hoc networks (VANETs). The need for decentralized authentication and authorization mechanisms that minimize or eliminate reliance on centralized authorities is becoming increasingly apparent. This drive toward decentralization is not merely a matter of architectural preference but a fundamental requirement for building robust, resilient, and scalable secure communication systems in the face of evolving threats and increasingly distributed environments. The exploration of novel cryptographic approaches that address both quantum resistance and decentralization is therefore a critical area of research in modern cryptography.

The initial signcryption schemes were primarily based on traditional public key infrastructure (PKI), inheriting the reliance on certificate authorities for key management and identity verification. However, the inherent limitations of PKI, particularly its centralized nature and certificate management overheads, motivated the development of more advanced signcryption paradigms. Identity-based signcryption (IBSC), introduced by Shamir in 1984 and later adapted to signcryption, has emerged as a promising alternative. IBSC eliminates the need for digital certificates by using a user’s identity (e.g., email address or username) as their public key. This simplifies key management significantly, as users do not need to obtain and manage certificates. Instead, a trusted private key generator (PKG) generates private keys for users based on their identities. While IBSC offers advantages in terms of key management simplicity, it introduces the key escrow problem, where the PKG has access to all users’ private keys. This key escrow issue is a significant concern in many applications, as it implies a centralized point of trust and potential for abuse. Tanksale [4] highlights vulnerabilities in identity-based signcryption schemes, demonstrating attacks against specific constructions, including Zhang et al.’s assertion signcryption scheme designed for decentralized autonomous environments. This underscores the importance of rigorous security analysis and the potential pitfalls of even seemingly decentralized identity-based approaches if not carefully designed. Furthermore, Tanksale [4] points out weaknesses in Yu et al.’s identity-based signcryption scheme in the standard model, showing its failure to achieve indistinguishability against chosen plaintext attacks. These findings emphasize the need for robust security proofs and careful consideration of attack vectors when designing and deploying identity-based signcryption schemes.

Certificateless signcryption (CLSC), proposed by Al-Riyami and Paterson in 2003, was developed to address the key escrow problem of IBSC while retaining the certificate-free nature of identity-based cryptography. CLSC eliminates the PKG’s ability to access users’ private keys by allowing users to generate partial private keys themselves, combined with a partial private key provided by a key generation center (KGC). This distributed key generation process mitigates the key escrow issue and enhances user autonomy. Xie et al. [5] propose a certificateless aggregate signcryption scheme for edge computing-based Internet of Vehicles (IoV), highlighting the relevance of CLSC in decentralized and resource-constrained environments. Their scheme aims to address the limitations of VANETs, such as network congestion and privacy leakage, by leveraging edge computing and certificateless cryptography. The scheme incorporates online/offline encryption and aggregate signcryption techniques to further enhance efficiency and security. Yang et al. [6] also focus on certificateless signcryption for VANETs, proposing a pairing-free online/offline scheme with batch verification for edge computing. They identify vulnerabilities in existing CLSC schemes, specifically in Xie et al.’s scheme, demonstrating its susceptibility to public key replacement attacks. Their proposed scheme aims to address these security concerns while also improving efficiency through online/offline signature and batch verification techniques. Rastegari et al. [7] revisit Luo and Wan’s certificateless signcryption scheme, pointing out errors in their construction and proposing a corrected and improved CL-SC scheme that is provably secure in the standard model. This work emphasizes the importance of rigorous security analysis and the ongoing refinement of CLSC schemes to ensure their robustness and practicality.

The security of signcryption schemes is typically analyzed using formal security models and provable security techniques. Provable security aims to demonstrate that a cryptographic scheme is secure under well-defined assumptions and against specific attack models. The random oracle model (ROM) is a widely used tool in provable security, where hash functions are modeled as ideal random oracles. While the ROM provides a useful framework for security analysis, it has been criticized for not accurately reflecting the behavior of real-world hash functions. Therefore, achieving provable security in the standard model without relying on random oracles is a desirable goal. Several of the reviewed papers, including Scheme [5], Scheme [6], Scheme [7], and Scheme [8], mention security proofs in the random oracle model (ROM). Scheme [4] and Scheme [7] emphasize provable security in the standard model, highlighting the stronger security guarantees offered by standard model proofs. Security proofs typically rely on computational hardness assumptions, such as the elliptic curve Diffie–Hellman (ECDH) problem or the computational Diffie–Hellman problem (CDHP). These assumptions are based on the presumed difficulty of solving certain mathematical problems, which underpins the security of the cryptographic schemes. Security analysis usually considers security properties such as confidentiality (indistinguishability against chosen ciphertext attacks, IND-CCA), unforgeability (existential unforgeability against adaptive chosen message attacks, EUF-CMA), and, sometimes, other properties, like anonymity or privacy preservation. Rigorous security analysis and provable security results are crucial for establishing confidence in the robustness and reliability of signcryption schemes.

The CFL certification system [9], proposed by researcher Chen Huaping and Professor Lü Shuwang, is an identity-based authentication mechanism. This system not only integrates the advantages of certificate-based and identity-based authentication but also effectively complements and enhances their respective limitations. The SM2 algorithm, independently designed in China as a commercial cryptographic algorithm, demonstrates advantages in resisting quantum computing attacks. Against this background, based on the limitations of existing signcryption schemes and drawing inspiration from the CFL authentication system, this research innovatively designs a decentralized signcryption scheme [10] implemented using the SM2 algorithm within the CFL framework. The security of this scheme has been rigorously proven using the random oracle model [11]. Finally, experiments demonstrate the scheme’s exceptional performance and effectiveness.

This paper makes significant contributions to the field of cryptography by addressing the limitations of existing signcryption schemes and proposing a novel decentralized signcryption scheme based on cryptography fundamental logics (CFL) and the SM2 algorithm. The key contributions of this paper are as follows:

Proposed decentralized signcryption scheme: The paper introduces a decentralized signcryption scheme that eliminates the need for a single certification authority (CA) to manage all keys. This scheme leverages the CFL authentication process, which achieves decentralized authentication and reduces the burden on certificate generation centers.

Security analysis: The security of the proposed scheme is rigorously analyzed under the random oracle model (ROM) and based on the complexity assumption of the elliptic curve Diffie–Hellman (ECDH) problem. This analysis ensures that the scheme provides strong resistance against quantum attacks and maintains confidentiality, unforgeability, public verifiability, non-repudiation, and forward secrecy.

Efficiency improvement: The theoretical analysis and experimental results demonstrate that the proposed scheme significantly improves computational and communication efficiency compared to traditional signcryption schemes. Specifically, the scheme reduces computational overheads by approximately 30% and communication overheads by approximately 20% in practical working environments.

The structure of this paper is organized as follows: Section 2 introduces preliminary knowledge; Section 3 presents the definition and specific details of the CFL-based decentralized signcryption scheme; Section 4 provides security proofs for the proposed scheme; and Section 5 analyzes the scheme’s efficiency through theoretical analysis and experimental evaluation. The paper concludes with final remarks.

## 2. Preliminary Knowledge

### 2.1. CFL: Cryptography Fundamental Logics

In response to current challenges in binding users with public keys in the identity authentication domain, the CFL (cryptography fundamental logics) system [12] has emerged as an innovative solution, ingeniously integrating the technical essence of both certificate-based and identity-based authentication to address existing problems.

In conventional certificate authentication systems, the relationship between public key (PK) and private key (SK) is typically formalized as PK = F(SK), where the public key, PK, is not directly associated with the user’s identity, ID. To establish the connection between PK and ID, a trusted third party (such as a certificate authority, CA) must be introduced to verify this relationship through certificate issuance. In current certificate authentication systems, trusted third parties play a central role, managing certificate application, issuance, verification, and revocation processes. As identity authentication application scenarios expand, the increasing burden on these third parties has become a constraint on overall system performance improvement.

In identity authentication mechanisms, the public key and user ID essentially form an inseparable entity, where the relationship between public and private keys can be further abstracted as SK = F(ID, MSK), with ID and PK considered equivalent elements. The key generation center (KGC) generates private keys for each user based on their ID and the system’s master secret key (MSK), implying that users do not have exclusive control over their private keys. As the identity authentication field increasingly emphasizes personal privacy protection, this approach, where KGC computes private keys for users, poses potential threats to system security, to some extent, challenging the overall security protection framework.

Researchers developed the certificate-free algorithm with CFL by combining the advantages of certificate- and identity-based authentication mechanisms. The framework involves generating two pairs of public–private keys: one identity-related and one randomly generated. The identity private key is used by the certificate authority (CA) for signing, while users maintain the random private key to sign their ID and request certificates, which are then stored securely in a UKey. Verifiers can derive both the identity public key and random public key from the user’s ID through specific algorithms, enabling decentralized verification without third-party intervention. This reduces the operational burden on certificate authorities while enhancing system efficiency and security. The CFL framework maintains identity authentication principles where identity public keys naturally link to user IDs, and the binding between user IDs and random public keys is secured through identity private key signatures and verification. By generating unique identity private keys for each user based on their ID, rather than using CA’s traditional single-key approach, the CFL system significantly improves security, enhances signature uniqueness, and strengthens the overall robustness of the authentication process.

### 2.2. Related Hard Problems

The signcryption scheme in this paper relies on the following hard problems.

**Definition 1.**  *Given an elliptic curve $E(F_{q})$ with elements $a,b\in F_{q}$, compute common parameters params={p,q,E,G,n}, where $G=(x_{G},y_{G})$ is the base point of the elliptic curve with order n. Given an elliptic curve E  with base point G  of order n , compute the common parameters abG. The elliptic curve Diffie–Hellman (ECDH) problem on this curve is defined as follows: Given aG, bG∈<G>, compute abG.*

**Definition 2.**  *Given an elliptic curve $E(F_{q})$with elements $a,b\in F_{q}$, compute common parameters params={p,q,E,G,n}, where $G=(x_{G},y_{G})$is the base point of the elliptic curve with order n. The discrete logarithm problem (DLP) on this curve is defined  [13] as follows: Given  aG∈<G>, find aG.*

## 3. CFL-Based Signcryption Scheme

This section delves into the core proposal of our research: the decentralized signcryption scheme built upon cryptography fundamental logics (CFL). The scheme represents a novel approach to signcryption by leveraging the unique advantages of CFL to address limitations in existing methods. It eliminates reliance on a central certification authority’s private key during public–private key generation, thereby enhancing security and decentralization.

### 3.1. Formal Definition

The scheme is abstracted into the following four algorithms:

Setup: A system initialization algorithm where users generate unique identifiers. The key generation center (KGC) receives a security parameter k as input and produces two crucial outputs: the master secret key (MSK) and system parameters (params). During this process, the MSK must be kept strictly confidential to ensure security, while the system parameters (params) are publicly available for widespread access and use.

The signcryption key pair is generated based on the user’s ID and public–private key fundamentals. A random public–private key pair ($P_{k}$, $S_{k}$) is generated, and the user’s ID along with the public key $P_{k}$ are submitted to the key generation center (KGC). The KGC verifies the authenticity and uniqueness of the submitted ID, then generates the identity public key$\;{ID}_{PK}=f\left(P_{k}\right)\;$and identity private key ${ID}_{SK}=f\left(S_{k}\right)$.

Signcrypt: The signcryption algorithm takes as the input the system parameters params, plaintext m, the recipient’s identity ${ID}_{B}$, and the sender’s private key ${SK}_{A}$, generating a ciphertext σ as the output. This algorithm can be represented as σ = Signcrypt (m, ${ID}_{SKA}$, ${ID}_{PKB}$).

Unsigncrypt: The unsigncryption algorithm takes as the input the ciphertext σ, the sender’s identity ${ID}_{A},\;$and the recipient’s private key ${SK}_{B}$, outputting either the plaintext message m or an error symbol “⊥” (indicating invalid or illegal ciphertext). This algorithm can be represented as m = Unsigncrypt(σ, ${ID}_{PKA}$, ${ID}_{SKB}$).

**Figure 1:**
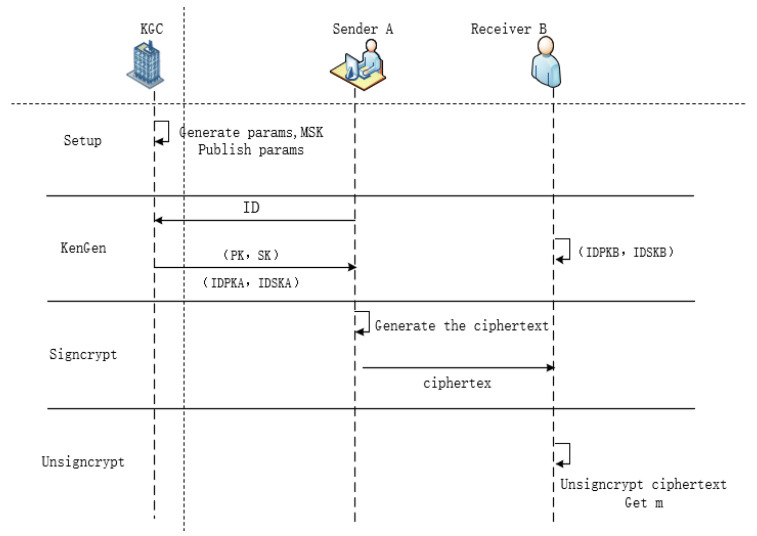
CFL-based signcryption algorithm.

These algorithms must satisfy the consistency principle of the signcryption scheme: if σ = Signcrypt(m, ${ID}_{SKA}$, ${ID}_{PKB}$), then m = Unsigncrypt(σ, ${ID}_{PKA}$, ${ID}_{SKB}$). As shown in Figure 1 below.

### 3.2. Scheme Design

Setup: Define two elements $a,b\in F_{q}\;$for the elliptic curve $E\left(F_{q}\right),$ compute common parameters params={p,q,E,G,n}, where p is a large prime number, E represents the elliptic curve defined over finite field $F_{q}$,$\;and\;G=(x_{G},y_{G})$ is the base point of order n on curve $E(F_{q})$.

KeyGen: Given a user’s ID, randomly generate a user’s public–private key pair ($P_{k}$, $S_{k}$). The KMC generates the identity private key IDSK using the ID and private key PASK and generates the verification identity public key IDPK using RAPK and the corresponding ID, where a relationship exists between the identity public key and the identity private key such that IDSK=IDPK·G.

Signcrypt: For user A (with identity ${ID}_{A}$, public key ${PAPK}_{A}$, identity private key ${IDSK}_{A}$) sending message m (length mlen) to user B:

1. Randomly select $k\in Z_{n}$;
2. Compute $C_{1}=kG=(x_{1},y_{1})$, and convert $C_{1}$ to bit string;
3. Compute e=Hash(Z||m),), and convert e to an integer, where Z is the hash value of the system parameters;
4. Compute $r=\left(e+x_{1}\right)mod\;n$; if r=0 or r+k=n, return to step (1) and repeat by randomly selecting a new k and recalculating r until the conditions are satisfied;
5. Compute$\;s=\left({\left(1+{IDSK}_{A}\right)}^{-1}\left(k-r{IDSK}_{A}\right)\right)mod\;n$; if s=0, return to step (1), select a new random number k, and recalculate the value of s until the conditions are satisfied;
6. Convert r, s to byte strings with lengths rlen, slen;
7. Compute $k\cdot {IDPK}_{B}=(x_{2},y_{2})$;
8. Compute klen=mlen+rlen+slen$t=KDF(x_{2}||y_{2},klen)$; if t is an all-0 bit string, return to step (1), iteratively select a new random number k, and recalculate t until the condition is met;
9. Compute $C_{2}=t⊕(m||r||s)$.
10. Generate ciphertext $\sigma =C_{1}||C_{2}$, and send σ to user B.

Unsigncrypt: User B receives ciphertext σ and performs the following operations:

1. Extract the bit string $C_{1}$ from ciphertext σ, and convert it to a point on the elliptic curve. Then, verify if point $C_{1}$ satisfies the elliptic curve equation. If not satisfied, terminate the operation;
2. Calculate $C_{1}\cdot {IDSK}_{B}=\left(x_{2}^{\prime },y_{2}^{\prime }\right);$
3. Calculateklen=mlen+rlen+slen, $t^{\prime }=KDF(x_{2}^{\prime }||y_{2}^{\prime },klen)$; if $t^{\prime }$ is an all-zero bit string, report error, and exit;
4. Calculate $m^{\prime }\left|\left|r^{\prime }\right|\right|s^{\prime }=t^{\prime }⊕C_{2}$;
5. Extract bit strings $r^{\prime }$, $s^{\prime },\;$ and convert them to integers. Verify if $r^{\prime }\in Z_{n}$, $s^{\prime }\in Z_{n}$. If not valid, report decryption failure, and exit;
6. Calculate $e^{\prime }=Hash(Z||m^{\prime })$, convert $e^{\prime }$ to an integer;
7. Calculate $u^{\prime }=\left(r^{\prime }+s^{\prime }\right)mod\;n$; if $u^{\prime }=0$, report decryption verification failure and exit;
8. Calculate the point $\left(x_{1}^{\prime },y_{1}^{\prime }\right)=s^{\prime }G+u^{\prime }\cdot {IDPK}_{A}$ on the elliptic curve;
9. Calculate $R=\left(e^{\prime }+x_{1}^{\prime }\right)modn$, and verify if $R=r^{\prime }$. If true, output plaintext m, and user B receives the message. Otherwise, report decryption verification failure, and exit.

## 4. Security Proof

This section rigorously examines the security properties of the proposed CFL-based signcryption scheme. The analysis is conducted under the random oracle model and is based on the hardness assumption of the elliptic curve Diffie–Hellman (ECDH) problem. The security of the scheme is evaluated against various attack vectors to ensure its robustness in real-world applications.

### 4.1. Security Definitions

The signcryption scheme must satisfy the following properties:

Confidentiality: Ensures that no information from the document can be obtained by an attacker through the ciphertext. The core concept is the computational infeasibility of decryption.

Unforgeability: Ensures that valid user signatures cannot be obtained by attackers. The core concept is the infeasibility of signature forgery.

Public verifiability: Ensures that signature verification has relative independence. This operation can be executed without requiring the recipient’s private key for verification. The core concept is the universal applicability of verification.

Non-repudiation: Ensures that once a sender chooses to send a command, they cannot deny their signcrypted message. The core concept is the undeniability of signatures.

Forward secrecy: Ensures the uniqueness of user private keys, meaning no method can recover the content of any previously signcrypted messages. The core concept is the non-recoverability of signatures.

**Definition 3.**  *The CFL-based signcryption scheme demonstrates IND-IBSC-CCA2 indistinguishability against adaptive chosen-ciphertext attacks if no polynomial-time adversary can win the game with significant advantage [14]. Here, IND-IBSC-CCA2 denotes the indistinguishability property of the signcryption scheme under adaptive chosen-ciphertext attacks (CCA2). Specifically, an adversary cannot distinguish between real and forged messages in their choices of ciphertext and plaintext. In this definition, indistinguishability means that even when an adversary can select ciphertexts and make queries across multiple stages (adaptive chosen-ciphertext attack), they cannot effectively distinguish between the original messages represented by any two ciphertexts. In other words, this definition ensures that even if an adversary can perform multiple operations on the ciphertexts, they still cannot obtain any useful information about the messages, thereby guaranteeing the message confidentiality of the system.*

1. Upon receiving security parameter k, challenger C executes the setup algorithm and transmits the resulting system parameters, params, to attacker A for subsequent use. During the query phase, adversary A performs the following operations:
2. Signcrypt query: Challenger C executes the key generation algorithm to generate the sender’s and receiver’s public/private key pairs, computes $\sigma =Signcrypt\left(m,{ID}_{PKA},{ID}_{PKB}\right),$ and sends σ to adversary A.
3. Unsigncrypt query: A submits a ciphertext σ and a sender’s public key ${ID}_{PKA}$ to challenger C. If the ciphertext is valid, C computes Unsigncrypt(σ, ${ID}_{PKA}$, ${ID}_{PKB}$) and returns message m to A; otherwise, the rejection symbol “⊥” is returned.
4. A generates two equal-length plaintexts, $m_{0}$ and $m_{1}$. C randomly selects u∈{0,1}, computes $\sigma =Signcrypt\left(m,{ID}_{PKA},{ID}_{PKB}\right),$ and sends the result σ to A.
5. During the guessing phase, similar to the search phase, A can make a polynomial-bounded number of queries.
6. At the end of the game, attacker A submits a predicted value b’ for b. If b’ matches b, then A is determined to have won the game.

A’s advantage is defined as $Adv(A)=|2\mathrm{Pr}\left[b^{\prime }=b\right]-1|$.

**Definition 4.**  *A CFL-based signcryption scheme is considered existentially unforgeable against adaptive chosen-message attacks (EUF-IBSC-CMA secure) if no polynomial-time adversary can win the game with significant advantage.*

1. Initialization phase: Challenger C accepts a security parameter k as input and executes the setup algorithm to generate the system parameter set params. Subsequently, C transmits the generated system parameters params to adversary A, enabling the adversary to utilize these parameters in subsequent operations.
2. As described in Definition 3, adversary A can make a polynomial-bounded number of queries to obtain the required information. These queries are constrained by polynomial bounds to ensure fairness and reasonability in the game.
3. Finally, adversary A outputs a new triple $\left(\sigma^{\prime },{IDPK}_{A},{IDSK}_{B}^{*}\right)$ A wins the game if this triple was not produced by the Signcrypt oracle and Unsigncrypt$\left(\sigma^{\prime },{IDPK}_{A},{IDSK}_{B}^{*}\right)$ does not return the symbol “⊥”.

A’s advantage is defined as their probability of winning.

### 4.2. Security Analysis

(1) Confidentiality: The confidentiality of this scheme is proven through Theorem 1.

**Theorem 1.**  *Under the random oracle model, if there exists an adversary A who complies with the IND-BCSC-CCA2 security model and can achieve a significant advantage ε within a specified time period t, meeting the success criteria described in Definition 3 (during which adversary A can perform at most $q_{k}$ key derivation function (KDF) queries, $q_{h}$ hash function queries, $q_{s}$ signcryption requests, and $q_{u}$ unsigncryption requests), then there exists a challenger C who can solve the ECDH problem with advantage $\varepsilon^{\prime }>\varepsilon \frac{2\varepsilon }{q_{k}+q_{s}}\;within\;time\;t^{\prime }\leq t+q_{k}t_{k}+q_{h}t_{h}+q_{s}t_{s}+q_{u}t_{u}$, where $t_{k}$ is the time for one KDF execution, $t_{h}$ is the time for one hash execution, $t_{s}$ is the time for one Signcrypt execution, and $t_{u}$ is the time for one Unsigncrypt execution.*

**Proof.**  Distinguisher C receives a randomly generated instance of the elliptic curve Diffie–Hellman (ECDH) problem as the input for analysis. Given $\left(G,P_{1},P_{2}\right)=(<G>,aG,bG)$, the goal is to compute abG; C runs A as a subroutine and plays the role of challenger in the IND-BCSC-CCA2 game. At the start of the game, C sets ${IDPK}_{B}=bG$, randomly selects $a^{\prime }\in Z_{n}^{*},$ and computes ${IDPK}_{A}=a^{\prime }G$. C then sends the system parameters $\left(G,n,{IDPK}_{B},{IDPK}_{A}\right)$ to challenger A. C maintains four initially empty lists: $L_{KDF}$, $L_{H}$, $L_{s}$, $L_{u}$, where $L_{s}$ simulates the signcryption oracle and $L_{u}\;$simulates the unsigncryption oracle.

KDF query: Upon receiving input $q_{KDF}=\left(x||y\right)$, C first checks if $q_{KDF}\;$exists in list $L_{KDF}$. If it exists, return $t_{i}$; if not, randomly select $t_{i}\in {\left\{0,1\right\}}^{klen}$, add $\left(q_{KDF},t_{i}\right)\;$to list $L_{KDF}$, and return $t_{i}$.

H query: Upon receiving a query, check if $\left(m,e_{i}\right)$ exists in list $L_{H}$. If found, return $e_{i};\;$if not found, randomly select $Z_{n}^{*}$, add $\left(m,e_{i}\right)\;$to $L_{H}$, and return $e_{i}$.

Signcrypt query:

Randomly select k from $Z_{n}^{*}$, compute $C_{1}=kG=(x_{1},y_{1})$;

Compute $e_{i}=H(Z||m)(H(Z||m)$(obtained from a hash query);

Compute $r=e_{i}+x_{1}$;

Compute $s={\left(1+a^{\prime }\right)}^{-1}\left(k-ra^{\prime }\right)$;

Compute $q_{KDF}=k\cdot {IDPK}_{B}$;

Compute $C_{2}=t_{i}⊕(m||r||s)$;

Return $\sigma =C_{1}||C_{2}$.

Unsigncrypt query:

Parse ciphertext σ into $C_{1}$ and $C_{2}$ components;

Compute $\left(x_{1},y_{1}\right)=C_{1}$; if $C_{1}\in L_{KDF}$, $t^{\prime }=t_{i}$, compute $m||r||s=t⊕C_{2}$, u=r+s, $\left(x_{1}^{\prime },y_{1}^{\prime }\right)=s^{\prime }G+u^{\prime }\cdot {IDPK}_{A}$;

If the equation $r=e+x_{1}^{\prime }$ holds, then return message m; otherwise, return an error symbol.

After a polynomial-bounded number of aforementioned queries, the adversary outputs two plaintext messages, $m_{0}$ and $m_{1},\;$of equal length. The challenger then computes $C_{1}^{\prime }=aG$, randomly selects $C_{2}^{\prime }{\left\{0,1\right\}}^{klen}$, constructs $\sigma =C_{1}^{\prime }||C_{2}^{\prime }$, and sends the challenge ciphertext σ to adversary A.

After the second round of queries, A’s inquiries remain identical to those in the first round. Upon successful completion of this simulation process, A submits a prediction value p’ as an estimate or inference of the original value p. If$\;p^{\prime }=p$, then C has solved the ECDH problem; otherwise, C has not solved the ECDH problem.

Assuming challenger C has previously input abG for a KDF query (meaning abG is stored in table) and C wins the above game with non-negligible advantage ε, then according to reference [4], the probability of the above event occurring is Pr≥2ε. The probability of randomly selecting from $L_{KDF}$ and obtaining exactly abG is at least $2\varepsilon /|L_{KDF}|$. Since $|L_{KDF}|\leq q_{k}+q_{s}$, the probability of C solving the ECDH problem is at least $\frac{2\varepsilon }{q_{k}+q_{s}}$. The computational time $t^{\prime }$ is the sum of all operational time costs incurred by challenger C and adversary A. Therefore,$t^{\prime }\leq t+q_{k}t_{k}+q_{h}t_{h}+q_{s}t_{s}+q_{u}t_{u}$.

The proof is complete. □

(2) Unforgeability: The unforgeability of the proposed scheme is established by the following Theorem 2.

**Theorem 2.**  *Under the assumption that the elliptic curve discrete logarithm problem (ECDLP) is computationally infeasible to solve and within the random oracle model, this scheme demonstrates strong resistance against forgery under adaptive chosen-message attacks.*

**Proof.**  Assume the signer receives a random instance of the elliptic curve discrete logarithm problem $\left(<G>,{IDPK}_{A}\right)$, where the objective is to find $a\in Z_{n}$ satisfying ${IDPK}_{A}=a\cdot G$.

Signature: The signer randomly selects $k\in Z_{n}$ and computes $C_{1}=kG=(x_{1},y_{1})$, $e\in Z_{n}$, $r=e+x_{1}$, $s={\left(1+{IDSK}_{A}\right)}^{-1}\left(k-r{IDSK}_{A}\right)$. The signature for message m is σ=(r,s).

Verification: According to reference [15], assume there exists an adversary A who can break the proposed signature scheme with parameters $\left(t,n_{sig},n_{h},\varepsilon \right)$, meaning that with access to at most $n_{sig}$ valid signatures and $n_{h}$ hash queries, A can successfully forge a valid signature within time t with a probability of no less than ε. Then, the adversary can obtain two signatures, $\left(r_{1},s_{1}\right)$ and $\left(r_{2},s_{2}\right)$, corresponding to the same random value $e^{\prime }$ within time $t^{*}$ with a probability of no less than $\varepsilon^{*}$ and compute $\frac{s_{2}-s_{1}}{s_{1}-s_{2}+r_{1}-r_{2}}$ satisfying $\frac{s_{2}-s_{1}}{s_{1}-s_{2}+r_{1}-r_{2}}\cdot G={IDPK}_{A}$.

Based on all the above analysis, under the assumption that the elliptic curve discrete logarithm problem (ECDLP) is computationally infeasible to solve and within the random oracle model, the signcryption scheme proposed in this paper maintains its unforgeability property even when subjected to adaptive chosen-message attacks. Thus, the security proof has been completed. □

(3) Public verifiability: Assume the ciphertext $\sigma =C_{1}||C_{2}\;$is generated by user A with user B as the recipient. The verifier can publicly verify whether the equation $\left(x_{1}^{\prime },y_{1}^{\prime }\right)=s^{\prime }G+u^{\prime }\cdot {IDPK}_{A}=sG+\left(r+s\right)G\cdot {IDSK}_{A}=kG=(x_{1},y_{1})$ holds. Thus, the scheme achieves public verifiability.

(4) Non-repudiation: Given the unforgeability property of the proposed scheme, if a sender has indeed signcrypted a message, they cannot deny the fact of having signcrypted that message. This ensures that the sender cannot repudiate their signed messages, thereby achieving non-repudiation.

(5) Forward security: In this scheme, the generation of public and private keys is independent of the certification authority’s private key, thus avoiding dependence on the certification authority’s private key during certificate generation and eliminating the risk of leakage or theft of the certification authority’s private key. Even if user A’s key is compromised or stolen, and even if private key information is inadvertently leaked, third parties cannot derive the session key from this information. Therefore, this scheme satisfies forward security.

### 4.3. Scyther: A Formal Tool for Security Protocol Analysis

Scyther is a formal tool for security protocol analysis. As an automated validation tool, it aims to identify vulnerabilities and attack paths in security protocols. It helps security researchers and protocol designers detect potential risks and improve protocol design. Scyther uses formal methods to analyze security protocols. It employs a formal modeling language for security protocols and performs automated analysis to uncover vulnerabilities and attack paths. Scyther uses model checking, symbolic execution, and simulation to analyze protocol security. It uses a modeling language based on security protocol description language (SPDL), which is a formal language specifically designed for security protocols. It describes messages, roles, and attack models in protocols. Scyther can detect common vulnerabilities in security protocols, such as authentication flaws, key distribution issues, and replay attack vulnerabilities. It can automatically analyze protocol models to detect design defects that may lead to security problems. Scyther can generate attack paths that attackers might exploit and attack sequences that may occur during protocol execution. This helps clarify potential security risks and vulnerabilities in protocols. Scyther offers a visual interface to display protocol models, vulnerability detection results, and attack paths. This allows users to intuitively understand analysis results and make further investigations and improvements. Scyther supports multiple security protocols, including TLS, SSH, IPSec, Kerberos, etc. In summary, Scyther is a powerful security protocol analysis tool that uses formal methods and automation to detect vulnerabilities and attack paths in security protocols. By using Scyther v1.1.3, security researchers and protocol designers can more comprehensively evaluate and improve the design of security protocols to enhance their security.

To analyze the security of a decentralized signcryption scheme based on CFL using the Scyther tool, first, the protocol process and message format of the scheme is converted into an SPDL model. Protocol roles, such as sender, receiver, and attacker, as well as message formats and processes, including the signcryption and unsigncryption stages and attacker interactions, are defined. Then, the SPDL model is input into the Scyther tool, analysis parameters like attacker capabilities are set, and the tool is run to generate protocol execution paths and detect potential attack paths. Finally, the analysis shows that the decentralized signcryption scheme based on CFL performs well in relation to security, effectively preventing issues like confidentiality breaches and signature forgeries. It also supports public key verification and non-repudiation and has forward security. Potential vulnerabilities, such as replay attacks, man-in-the-middle attacks, and public key replacement attacks, can be mitigated by adding timestamps, enhancing key exchange security, and strictly managing public keys. This scheme holds promise for high-security applications like V2X and IoT.

## 5. Performance Analysis

This section provides a comprehensive evaluation of the newly proposed signcryption scheme based on computational complexity and communication costs [16].

### 5.1. Computational Overhead

In the simulation process, the system adopts the C/C++ library of high-precision integer operation and rational arithmetic and integrates the encryption algorithm library based on pairing theory. In the simulation, the execution time of the bilinear pairing operation is expressed as $t_{bp}=6.574\;ms$, the execution time of scalar multiplication on bilinear pairs is expressed as $t_{bpm}=2.123\;ms$, the execution time of scalar multiplication on elliptic curves is expressed as $t_{m}=0.576\;ms$, the execution time of point addition on elliptic curves is expressed as $t_{a}=0.02\;ms$, the execution time of modular exponentiation is expressed as $t_{ex}=0.249\;ms$, and the execution time of hashing operations is expressed as $t_{h}=0.002\;ms$.

**Figure 2:**
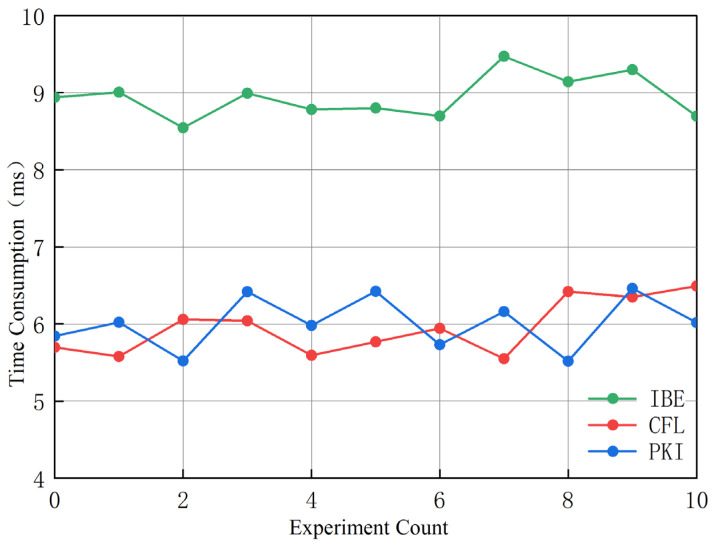
Time consumption comparison.

Table 1 compares the performance differences between our newly proposed scheme and several signcryption schemes. The proposed scheme shows competitive performance compared to the referenced schemes in Figure 2. It has a signcryption time of 1.738 ms, unsigncryption time of 3.566 ms, and a total overhead of 5.304 ms. Compared to other schemes, our scheme has a lower computational overhead and a shorter execution time. These performance metrics enhance its applicability in real-world scenarios, especially in environments with limited computational resources.

**Table 1:** Comparison between our scheme and other signcryption schemes.

Scheme	Signcryption (ms)	Unsigncryption (ms)	Total Overhead (ms)
Reference [17]	$3t_{m}+t_{a}+t_{ex}+3t_{h}$	$3t_{m}+3t_{a}+3t_{h}+t_{bp}$	$6t_{m}+4t_{a}+t_{ex}+6t_{h}+t_{bp}$
Reference [18]	$6t_{m}+4t_{a}+5t_{h}$	$3t_{m}+1t_{a}+7t_{h}+5t_{bp}$	$9t_{m}+5t_{a}+12t_{h}+5t_{bp}$
Reference [19]	$4t_{ex}$	$2t_{ex}+2t_{bp}$	$6t_{ex}+2t_{bp}$
Reference [20]	$7t_{m}+t_{h}$	$2t_{h}$	$3t_{h}$
Reference [21]	$5t_{m}+t_{bp}$	$t_{ex}+t_{bp}$	$2t_{bp}+5t_{m}+t_{ex}$
Proposed Scheme	$3t_{m}+5t_{h}$	$6t_{m}+5t_{a}+5t_{h}$	$9t_{m}+5t_{a}+10t_{h}$

For experimental verification, the test program was built using the open-source cryptographic library Bouncy Castle and written in Java programming language. The experimental laptop configuration included the following: Intel(R) Core(TM) i7-8550U processor (Intel Corporation, Santa Clara, CA, USA), 8 GB RAM, running Windows 11 64-bit operating system. The average efficiency overhead of the CFL-based signcryption scheme was approximately 6.32 ms, the PKI-based signcryption scheme was about 5.79 ms, and the IBE-based signcryption scheme was around 9.63 ms. This demonstrates that the time complexity of CFL-based and PKI-based signcryption schemes is similar, while the IBE-based signcryption scheme has lower efficiency due to higher computational overheads, consistent with the theoretical analysis.

### 5.2. Communication Overhead

Since certificate registration and verification occur independently, this paper discusses the communication overhead for both phases separately, ignoring the communication gap between certificate authentication and registration. Conclusions are drawn by comparing our scheme with two other mainstream schemes.

The registration phase involves data users submitting identity information and registration requests to the certification authority and the certification authority generating and returning certificates. The certificate authentication phase involves data users sending certificates to the cloud data center and the cloud data center verifying and returning the authentication results. In the authentication system’s communication interaction process, the core components include device-to-device communications and remote communications between devices and authentication authorities. Here, RV represents the cost of a single short-range communication between device nodes, while RC represents the cost of a single remote communication between devices and the certification center. Table 2 details the comparative analysis of the communication costs for three different schemes.

As shown in Table 2, during the registration phase, the PKI scheme, IBE scheme, and our proposed CFL scheme have identical communication overheads, each requiring two RC sessions. During the authentication phase, since our scheme’s authentication process does not require third-party certification authority participation, it requires two fewer device-to-certification center sessions compared to the PKI and IBE schemes. The PKI and IBE schemes have identical communication overheads, both requiring two device-to-certification center sessions and two device-to-device sessions. The comparison demonstrates that our proposed scheme effectively reduces the communication phase overhead.

To verify the aforementioned theoretical analysis, the experimental setup was designed as follows: A Tencent Cloud server in Shanghai was deployed with the Ubuntu 20.04 system (Server C) as the remote node, equipped with Intel Xeon Platinum 8255C CPU and a Tencent VirtIO network card (Tencent Technology (Shenzhen) Company Limited, Shenzhen, China). In addition, two Windows 10 laptops (A and B) were configured in adjacent campus buildings as local devices, simulating the realistic network layout of the certification authority and communication entities. The Microsoft PsPing tool was then utilized to conduct continuous inter-device access tests, meticulously recording the number of transit nodes for each communication and average round-trip delay to thoroughly investigate network performance. The average round-trip latency measured by the testing tool represents the transmission delay for data packets traveling from the source to the destination and back to the source in the network. This metric is primarily influenced by the number of routing hops and real-time network load conditions and can be considered as a communication cost independent of device performance.

**Figure 3:**
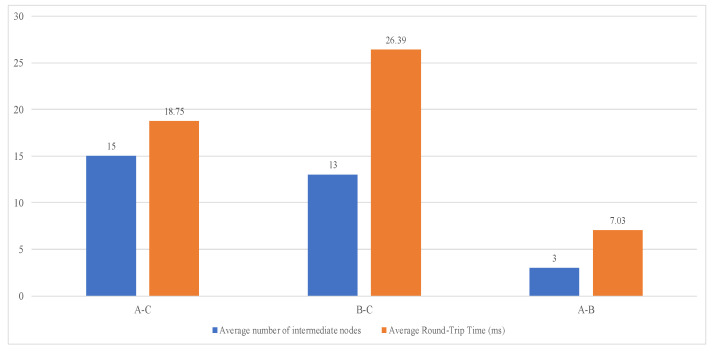
Remote access time consumption comparison.

In this experiment, laptops A and B simulated the sender and receiver, respectively, with access to remote server C simulating the certificate verification process. The average delay RC was used to record remote communication (C-end) latency, while interactions between A and B simulated short-distance communication, with the average delay recorded as RV. The experimental data packet size followed SSL/TLS protocol specifications, set at 500 bytes, with 100 inter-access attempts per group [22]. The final experimental results are shown in Figure 3.

Based on these experimental results, the short-range communication overhead can be estimated as RV ≈ 7.03/2 = 3.515 ms and the remote communication overhead as RC ≈ (18.75 + 26.39)/4 = 11.285 ms. Calculations show that the PKI and IBE schemes require approximately 52.17 ms of communication overheads during the authentication phase, while our proposed scheme requires only about 29.60 ms due to its center-free verification phase. This experimental design did not cover practical challenges such as packet loss, network congestion, connection establishment requests, and data caching. In real-world application scenarios, centralized authentication mechanisms like PKI or IBE might incur higher communication costs. In comparison, our proposed scheme demonstrates advantages in communication overheads, meeting millisecond-level security response requirements, highlighting its significant potential and value in practical applications.

## 6. Conclusions

In this study, a decentralized signcryption scheme based on the cryptography fundamental logics (CFL) framework was proposed. This scheme represents a significant departure from traditional signcryption schemes, as it does not rely on the certification authority’s private key during the public–private key generation process. By eliminating this dependency, the proposed scheme effectively resolves the risk of private key leakage from the certification authority and achieves robust decentralized characteristics. This is a crucial advancement in cryptographic security, as it reduces the potential attack surface and enhances the overall security posture.

Security analysis of the proposed scheme was conducted under the random oracle model, a widely accepted framework for analyzing cryptographic protocols. The security of the scheme was rigorously proven under the hardness assumption of the elliptic curve Diffie–Hellman (ECDH) problem. This mathematical foundation provides a strong theoretical guarantee of the scheme’s security against various types of attacks.

Comparative analysis with existing traditional signcryption schemes revealed that the proposed scheme demonstrates significant advantages in both communication and computational efficiency [23]. These improvements are particularly noteworthy in practical applications where resources are limited and performance is critical. The experimental results further corroborate these findings, indicating that the proposed scheme not only offers enhanced security but also maintains superior efficiency, making it highly suitable for implementation in real-world scenarios.

Future research should focus on designing even more efficient CFL-based signcryption schemes and exploring their applications in diverse practical working environments [24]. This could include optimizing the cryptographic algorithms used, developing new protocols for specific application scenarios, and conducting extensive empirical studies to validate the scheme’s performance in different contexts. Additionally, further research could explore the integration of this scheme with other emerging technologies, such as blockchain, to create more secure and decentralized systems.

## Figures and Tables

**Table 2:** Communication overhead comparison of different schemes.

Scheme	Registration Phase	Authentication Phase	Total Communication Overhead
PKI	2RC	2RC + 2RV	4RC + 2RV
CFL	2RC	2RV	2RC + 2RV
IBE	2RC	2RC + 2RV	4RC + 2RV

## Data Availability

The data that support the findings of this study are not publicly available due to privacy concerns. Requests for access to the data should be directed to the corresponding author.

## References

[1] Zheng Y. Digital Signcryption or How to Achieve Cost(Signature & Encryption) << Cost(Signature) + Cost(Encryption). Proceedings of the Annual International Cryptology Conference.

[2] Yu H.F., Qiao Y.F., Meng R. (2023). Quantum-resistant attribute-based threshold ring signcryption scheme for blockchain finance. Inf. Netw. Secur..

[3] Ke-Zhen Z., Jing-Qiang L., Wei W., Yong L., Guang-Zheng L., Zhen-Ya L. (2024). Research on Two-Party SM2 Threshold Signature Schemes with a Blind Cooperative Server. J. Cryptologic Res..

[4] Wang X., Qian H. Attacks against Two Identity-Based Signcryption Schemes. Proceedings of the Second International Conference on Networks Security, Wireless Communications and Trusted Computing.

[5] Xie Z., Chen Y., Ali I., Pan C., Li F., He W. (2023). Efficient and Secure Certificateless Signcryption Without Pairing for Edge Computing-Based Internet of Vehicles. IEEE Trans. Veh. Technol..

[6] Yang W., Cao P., Zhang F. (2025). A Secure Pairing-Free Certificateless Online/Offline Signcryption Scheme with Batch Verification for Edge Computing-Based VANETs. IEEE Trans. Veh. Technol..

[7] Rastegari P., Susilo W., Dakhlalian M. (2019). Efficient Certificateless Signcryption in the Standard Model: Revisiting Luo and Wan’s Scheme from Wireless Personal Communications (2018). Comput. J..

[8] Ullah I., Khan M.A., Kumar N., Abdullah A.M., AlSanad A.A., Noor F. (2023). A Conditional Privacy Preserving Heterogeneous Signcryption Scheme for Internet of Vehicles. IEEE Trans. Veh. Technol..

[9] Xie Y.F., Zhao D.D., Shi L.Y. (2024). Signature Authentication Scheme for Decentralized Industrial Control System Based on CFL. Comput. Syst. Appl..

[10] Zhang Y., Wang Z., Qin T. (2024). An Efficient Signature and Encryption Scheme Based on SM2. Inf. Secur. Res..

[11] Brendel J., Cremers C.J.F., Jackson D., Zhao M. The Provable Security of Ed25519: Theory and Practice. Proceedings of the 2021 IEEE Symposium on Security and Privacy (SP).

[12] Chen H.P., Fan X.B., Lv S.W. (2013). Certificate Authentication System CFL Based on Identity.

[13] Hu R.L., Li W.J., Jiang H., Zhang X.R. (2019). Certificateless aggregate signcryption scheme based on discrete logarithm. Inf. Netw. Secur..

[14] Qin H., Pan Y., Fan X., Wang H. (2016). Provably Secure Analysis of CFL. Inf. Secur. Res..

[15] Ohta K., Okamoto T. On Concrete Security Treatment of Signatures Derived from Identification. Proceedings of the Annual International Cryptology Conference.

[16] Hussain S., Ullah I., Khattak H., Khan M.A., Chen C.M., Kumari S. (2021). A lightweight and provable secure identity-based generalized proxy signcryption (IBGPS) scheme for Industrial Internet of Things (IIoT). J. Inf. Secur. Appl..

[17] Yang Y., He D., Vijayakumar P., Gupta B.B., Xie Q. (2022). An Efficient Identity-Based Aggregate Signcryption Scheme With Blockchain for IoT-Enabled Maritime Transportation System. IEEE Trans. Green Commun. Netw..

[18] Yang Y., Zhang L., Zhao Y., Choo K.K.R., Zhang Y. (2022). Privacy-Preserving Aggregation-Authentication Scheme for Safety Warning System in Fog-Cloud Based VANET. IEEE Trans. Inf. Forensics Secur..

[19] Karati A., Islam S.H., Biswas G.P., Bhuiyan M.Z.A., Vijayakumar P., Karuppiah M. (2018). Provably Secure Identity-Based Signcryption Scheme for Crowdsourced Industrial Internet of Things Environments. IEEE Internet Things J..

[20] Nkenyereye L., Liu C.H., Song J.S. (2019). Towards secure and privacy preserving collision avoidance system in 5G fog based Internet of Vehicles. Future Gener. Comput. Syst..

[21] Deng L. (2020). Anonymous certificateless multi-receiver encryption scheme for smart community management systems. Soft Comput..

[22] Lan S.B., Li F.X., Shi L.Y. (2023). Authentication communication scheme for industrial control system based on CFL. J. Comput. Appl..

[23] Ashok K., Gopikrishnan S. (2024). A Hybrid Secure Signcryption Algorithm for data security in an internet of medical things environment. J. Inf. Secur. Appl..

[24] Hussain S., Ullah S.S., Uddin M., Iqbal J., Chen C.-L. (2022). A Comprehensive Survey on Signcryption Security Mechanisms in Wireless Body Area Networks. Sensors.

